# Longitudinal impacts of pubertal timing and weight status on adolescent Internet use: Analysis from a cohort study of Taiwanese youths

**DOI:** 10.1371/journal.pone.0197860

**Published:** 2018-05-24

**Authors:** Meng-Che Tsai, Carol Strong, Wan-Ting Chen, Chih-Ting Lee, Chung-Ying Lin

**Affiliations:** 1 Department of Pediatrics, National Cheng Kung University Hospital, College of Medicine, National Cheng Kung University, Tainan, Taiwan; 2 Department of Public Health, National Cheng Kung University Hospital, College of Medicine, National Cheng Kung University, Tainan, Taiwan; 3 Department of Counseling and Guidance, College of Education, National University of Tainan, Tainan, Taiwan; 4 Department of Family Medicine, National Cheng Kung University Hospital, College of Medicine, National Cheng Kung University, Tainan, Taiwan; 5 Department of Rehabilitation Sciences, Faculty of Health and Social Sciences, The Hong Kong Polytechnic University, Kowloon, Hong Kong; East Tennessee State University, UNITED STATES

## Abstract

**Aim:**

To investigate the longitudinal impacts of pubertal timing and weight status on Internet use in adolescents.

**Methods:**

Three waves of data on a longitudinal cohort of 7^th^ grade students (N = 2430) were retrieved from the Taiwan Youth Project. Univariate and multivariate regression models were applied using crude and adjusted odds ratios (OR) with 95% confidence intervals (CI) to examine the concomitant impacts of pubertal timing and weight status on adolescent Internet use.

**Results:**

The dataset identified 210 (8.7%) students using the Internet for more than 20 hours/week, and 81 (3.3%) were viewing pornographic material online. Early maturing and thin-weight adolescents were at 35% and 46% increased risks of spending long hours on Internet use, respectively. While early puberty was associated with online pornography viewing among males (adjusted OR 1.84, 95% CI 1.04–3.28), early puberty was contrarily a protective factor against online gaming in females (adjusted OR 0.59, 95% CI 0.36–0.96).

**Conclusion:**

Early puberty was found to be positively related to adolescent Internet use. Appropriate health education and guidance regarding Internet use should be provided to those with different developing needs.

## Introduction

In an era of digitized technology, the Internet has rapidly evolved into a new platform of information, communication and entertainment, but it can also be a potentially hazardous medium due to unverified or uncensored content, particularly for young people [1]. A recent European report estimates that around 70% of adolescents have Internet access at home and spend an average of 2 hours daily online [2]. Problematic Internet users, like those who are excessively playing online games, frequently checking emails, or viewing pornography, could spend twice that amount of time [2]. This figure is comparative in high school students in Taiwan, where academic achievement is highly stressed and online gaming is highly developed and mainly focuses on this particular age group [2–4].

Ongoing research has linked excessive Internet use and weight status in adolescents, but the results are inconsistent [5–8]. In China, obesity has been found to be more prevalent in adolescents with Internet addiction assessed with self-reported questionnaires [7]. A similar finding that was reported in a mixed sample recruited from several European countries showed that heavier Internet use, defined as more than 2 hours/day, was associated with overweight/obesity [8]. However, an Australian study failed to demonstrate such a positive association between weight status and computer use or video gaming after controlling the time for television viewing [5]. A longitudinal study with a 2-year follow-up further indicated that the risk of obesity in Swiss adolescents was mostly influenced by weight status at the baseline rather than daily hours of Internet use [6]. Variations in assessments of Internet use may explain the discrepancy of results. Contextual differences in social values and adolescent lifestyles may also contribute to the extent of correlation between weight status and Internet use. Moreover, previous research has shown a U-shaped association between Internet use and adolescent psychosocial problems [9], which indicates that the purposes or content of online activities may underlie the link between the intensity of Internet use and an unhealthy weight status, if there is one. Given that the inferential relationship between weight status and the Internet use could be bi-directional, further research attention should be given to analyze the effect of weight status on adolescent Internet use.

Pubertal hormones surge and contribute in part to the organization of neural circuits during adolescence, a critical time of neurocognitive and psychosocial development [10]. As such, sexual maturity is another important issue when addressing adolescent social behaviors, including Internet use. Previous research on this issue has substantially investigated exposure to pornography during the pubertal stage [11–13], with a few specifically examining the impact of pubertal timing [14–16]. Conflicting results also exist, and gender plays a part in the association between early puberty and online pornography viewing. For example, Swedish boys with advanced scores in subjective ratings on their own pubertal development reported higher rates of access to online pornography [14]. However, no differences were found when examining pornography exposure among American adolescents when they were asked to compare their sexual maturity to peers [16]. Further, a Swiss sample demonstrated that only early maturing females with perceived early pubertal status had more online pornography exposure [15]. These findings were mainly derived from studies conducted in Western societies. Little is known about how pubertal timing impacts adolescent Internet use in an East Asian context, where cross-cultural validation research may provide further insights into the pubertal effects on adolescent online behaviors and thus source adolescent developmental guidance and public policy implementation locally.

According to the literature portrayed above, this study aims to concomitantly consider the impacts of pubertal timing and weight status on adolescent Internet use, because both early puberty and obesity are biologically correlated [17]. We hypothesized that early sexual maturation and obesity might contribute to heavy Internet use. The association between the leisure purposes for Internet use and pubertal timing (PT) and weight status (WS) were also examined in this longitudinally followed cohort of Taiwanese youths.

## Methods

### Participants

We used data from the Taiwan Youth Project (TYP), which is a publicly accessible longitudinal survey conducted by the Institute of Sociology, Academia Sinica, Taiwan (https://srda.sinica.edu.tw). The detailed procedures and survey items of TYP are described elsewhere [18]. In brief, this project used a multi-stage cluster sampling design to survey 7^th^ and 9^th^ graders among 40 schools in northern Taiwan. Participants were informed of this study and gave their consents only after completing the questionnaire. A baseline interview took place in 2000, with annual follow-up surveys, which are still ongoing, and the datasets are publicly available (www.typ.sinica.edu.tw). The final analysis in the present study included only the 7^th^ graders, who completed all the relevant questions from the first 3 waves of the database. This study was approved by the Institutional Review Board of the National Cheng Kung University Hospital (A-ER-103-009).

### Measures

#### Pubertal timing

The Chinese version of Pubertal Developmental Scale (PDS), which has been validated and corresponded to the Tanner staging of puberty in the Taiwanese population, has been used to measure the subjective evaluation of pubertal changes in adolescent health research [19]. The PDS items, including growth spurts, body hair development, skin changes, breast growth/voice change, and menarche/facial hair growth, were assessed at the 7^th^ grade (Wave 1). Except for menarche, which was a dichotomous item (“yes” or “no”), all other items were rated using a 4-point Likert scale. We summarized the PDS scores with a higher score representing an earlier timing of sexual maturation. Further, we standardized the scores within same-gender cohorts, and we classified the participants into three PT groups: early-puberty (more than 1 standard deviation [SD] above), on-time puberty (within 1 SD either way), and late-puberty (more than 1 SD below) [19].

#### Weight status

Based on self-reported body heights and weights, body mass index (BMI) was calculated and used to classify the same-gender participants into 4 groups: thin weight (more than 1 SD below), normal weight (within 1 SD either way), overweight (between 1 and 2 SDs above), and obese (more than 2 SDs above). The cutoff BMI values for each group were comparable to those defined previously for the Taiwan adolescent population [20]. We included their WS at the 7^th^ (Wave 1) and 9^th^ grade (Wave 3) in the analysis. In order to calculate the BMI changes between W1 and W3, we standardized the BMI results within the same-gender group before obtaining the differences in the standardized BMI scores (BMI-SDS).

#### Internet use

The amount of time and purposes of Internet use assessed at the 9^th^ grade were the major outcome variables under investigation in this study. The weekly length of time spent on the Internet was categorized into 6 groups: “less than 1 hour”, “1–2 hours”, “3–5 hours”, “6–10 hours”, “11–20 hours”, and “more than 20 hours”. For analytic purposes, we transformed the amount of time into an ordinal scale ranging from 1 (less than 1 hour) to 6 (more than 20 hours) [12]. Meanwhile, the purposes of Internet use that we particularly focused on were the non-academic activities, such as online chatting, online gaming, non-academic browsing, and pornography viewing. The answers were dichotomous with only a “yes” or “no” option.

#### Covariates

Monthly family income was used as a proxy for the socioeconomic status of the subjects. This covariate was subdivided into three levels: “New Taiwan Dollar (NTD) 30000 or less”, “NTD 30001–60000”, and “NTD 60001 or more”.

### Statistical analysis

Demographic characteristics of the subjects were summarized using descriptive analysis. The correlates of adolescent internet use were examined using analysis of variance (ANOVA), independent *t* test, and χ^2^ test, as appropriate. For example, a χ2 test for linear-by-linear association was applied to evaluate the trends of the amount of time and purposes of Internet use across different PT and WS groups. Also, we compared the BMI-SDS changes among the groups with different amount of time and purposes of Internet use using ANOVA and independent t test, respectively. Further adjusting for family monthly income, we described the differential impacts of PT, WS, and BMI-SDS changes on the amount of time of Internet use in univariate and multivariate ordinal logistic regression models. Although weight status at Wave 1 and 3 were correlated (Spearman σ = 0.67, *p*<0.001), we kept them in multivariate models to elaborate the effects of recent and remote weight status [6]. Likewise, univariate and multivariate binary logistic regression analyses with the genders stratified were applied to analyze the associated factors of different leisure purposes of Internet use. All of the statistical analyses were conducted using SPSS17.0 (SPSS Inc., Chicago, IL, USA).

## Results

A total of 2430 adolescents corresponding to 90.3% of the recruited 7^th^ graders, 1241 (51.1%) of which were males, were included in the final analysis (Table 1). The original PDS scores were higher among females (mean 11.35 [±2.11]) than males (mean = 9.47 [±2.25]). On the other hand, the mean of the males’ BMI was greater than that of the females’ both at Waves 1 and 3. In general, males had more frequent uses of the Internet than females. More than one third of female participants were using the Internet for less than one hour a week, whereas 15% of male counterparts reported more than 20 hours spent online. The purposes of Internet uses differed by gender. In males, 70.1% and 6.2% reported use for online gaming and pornography viewing, respectively. Meanwhile, female participants usually used the Internet for chatting (43.7%) and website browsing (55.6%).

**Table 1:** Demographic characteristics of subjects (N =2430).

	Male (N = 1241)	Female (N = 1189)	*p*-value
Age	13.28 (±0.45)	13.30 (±0.46)	0.177
Pubertal developmental scale at W1	9.47 (±2.25)	11.35 (±2.11)	<0.001
Body mass index at W1	20.43 (±4.19)	19.51 (±3.18)	<0.001
Body mass index at W3	21.04 (±3.98)	20.32 (±3.15)	<0.001
Family monthly income (NTD)			0.827
< 30000	203 (18.0)	192 (16.1)	
30000–60000	487 (43.2)	481 (40.5)	
>60000	438 (38.8)	409 (34.4)	
Internet use at W3			<0.001
< 1 hour/week	272 (21.9)	439 (36.9)	
1–2 hours/week	215 (17.3)	332 (27.9)	
3–5 hours/week	262 (21.1)	244 (20.5)	
6–10 hours/week	159 (12.8)	107 (9.0)	
11–20 hours/week	147 (11.8)	43 (3.6)	
> 20 hours/week	186 (15.0)	24 (2.0)	
Leisure purpose of Internet use at W3			
Chatting	340 (27.4)	519 (43.7)	<0.001
Gaming	870 (70.1)	256 (21.5)	<0.001
Non-academic browsing	499 (40.2)	661 (55.6)	<0.001
Pornography viewing	77 (6.2)	4 (0.3)	<0.001

Data are presented as n (%) or mean (± standard deviation). Comparison of variables between male and female participants was examined by student t test and χ2 test accordingly. NTD represents New Taiwan Dollar; W1, Wave 1; W3, Wave 3.

Table 2 summarizes the associations between the amount of time for Internet use and PT and WS. A higher prevalence of excessive Internet use, more than 20 hours/week, was noted among those with early maturation (11.7%) at W1 and obesity at W3 (12.3%). However, the trends analysis was not significant between PT and WS and Internet use. In the univariate model with family income adjusted, those who were early-maturing (odds ratio [OR] 1.36, 95% confidence interval [CI] 1.11–1.66) and thin-weight (OR 1.39, 95% CI 1.09–1.77) were at a higher risk of increased length of Internet use. This association remained significant even in the multivariate analysis with controlling potential correlates.

**Table 2:** Univariate and multivariate association between time of Internet use and pubertal timing and weight status.

	Internet use (hours/week)							Univariate	Multivariate
	< 1	2–3	3–5	5–10	11–20	>20	p-value	OR (95% CI)	aOR (95% CI)
Pubertal timing, N (%)									
Early puberty	98 (24.9)	89 (22.6)	78 (19.8)	45 (11.5)	37 (9.4)	46 (11.7)	0.341	1.36 (1.11–1.66)	1.35 (1.10–1.66)
On-time puberty	489 (30.6)	371 (23.2)	334 (20.9)	166 (10.4)	118 (7.4)	122 (7.6)		Reference	Reference
Late puberty	124 (28.4)	87 (19.9)	94 (21.5)	55 (12.6)	35 (8.0)	42 (9.6)		1.18 (0.96–1.43)	1.15 (0.94–1.41)
BMI at W1, N (%)									
Thin weight	79 (27.9)	67 (23.7)	63 (22.3)	28 (9.9)	23 (8.1)	23 (8,1)	0.409	1.07 (0.84–1.33)	0.89 (0.67–1.20)
Normal weight	539 (29.9)	400 (22.2)	375 (20.8)	201 (11.2)	131 (7.3)	154 (8.6)		Reference	Reference
Overweight	64 (27.4)	54 (23.1)	47 (20.1)	24 (10.3)	24 (10.3)	21 (9.0)		1.08 (0.84–1.38)	1.07 (0.76–1.51)
Obesity	29 (26.6)	26 (23.9)	21 (19.3)	13 (11.9)	11 (10.1)	19 (8.3)		1.12 (0.78–1.60)	0.96 (0.52–1.79)
BMI at W3, N (%)									
Thin weight	72 (27.5)	47 (17.9)	50 (19.1)	41 (15.6)	23 (8.8)	29 (11.1)	0.795	1.39 (1.09–1.77)	1.46 (1.08–1.95)
Normal weight	544 (29.6)	428 (23.4)	386 (21.0)	193 (10.5)	136 (7.4)	149 (8.1)		Reference	Reference
Overweight	64 (29.4)	47 (21.6)	50 (22.9)	19 (8.7)	20 (9.2)	18 (8.3)		1.02 (0.79–1.32)	0.99 (0.69–1.42)
Obesity	31 (27.2)	25 (21.9)	20 (17.5)	13 (11.4)	11 (9.6)	14 (12.3)		1.24 (0.87–1.76)	1.26 (0.67–2.36)
ΔBMI-SDS, Mean (SD)	0.004 (.557)	0.010 (.554)	0.008 (.463)	-0.047 (.451)	-0.032 (.495)	0.031 (.574)	0.554	0.95 (0.83–1.10)	0.98 (0.81–1.18)

OR represents odds ratio; CI, confidence interval; aOR represents adjusted odds ratio; BMI-SDS, standardized score of body mass index; SD, standard deviation; W1, Wave 1; W3, Wave 3. Family monthly income was adjusted in univariate and multivariate ordinal logistic regression analysis.

In order to investigate the differential effects of gender on the purpose of Internet use, we stratified our analysis by gender. Among males (Table 3), we found that there were increasing trends between PT and non-academic browsing (*F* = 11.53, *p* = 0.001) and pornography viewing (*F* = 4.18, *p* = 0.04), as well as those found between WS at W1 and online gaming (*F* = 4.20, *p* = 0.04) and pornography viewing *(F* = 7.12, *p* = 0.008). In multivariate models, we found a nearly 85% increase of risk (adjusted OR 1.84, 95% CI 1.04–3.28) for viewing pornography among early-maturing males. Contrarily, early maturing girls were protected against online gaming (adjusted OR 0.59, 95%CI 0.36–0.96), while thin-weight girls had a higher likelihood of online gaming (adjusted OR 2.50, 95% CI 1.39–4.48) as compared to reference groups (Table 4).

**Table 3:** Association between the purposes of Internet use and pubertal timing and weight status among male adolescents.

	Chatting		Gaming		Non-academic browsing		Pornography viewing	
	N (%)	aOR (95% CI)	N (%)	aOR (95% CI)	N (%)	aOR (95% CI)	N (%)	aOR (95% CI)
Pubertal development								
Early puberty	69 (29.5)	1.01 (0.72–1.41)	163 (69.7)	0.89 (0.63–1.24)	**110 (47)**	1.17 (0.86–1.61)	**23 (9.8)**	1.84 (1.04–3.28)
On-time puberty	204 (28.3)	Reference	508 (70.5)	Reference	**296 (41.1)**	Reference	**39 (5.4)**	Reference
Late puberty	67 (23.4)	0.72 (0.51–1.02)	199 (69.6)	0.93 (0.68–1.28)	**93 (32.5)**	0.76 (0.56–1.03)	**15 (5.2)**	1.07 (0.55–2.08)
BMI at W1								
Low weight	32 (22.9)	0.83 (0.50–1.39)	**88 (62.9)**	0.72 (0.45–1.14)	43 (30.7)	0.71 (0.46–1.13)	**5 (3.6)**	0.67 (0.22–2.00)
Normal weight	251 (27.6)	Reference	**643 (70.8)**	Reference	373 (41.1)	Reference	**55 (6.1)**	Reference
Overweight	33 (25.4)	0.93 (0.51–1.69)	**85 (65.4)**	0.83 (0.47–1.46)	60 (46.2)	1.16 (0.68–1.99)	**8 (6.2)**	1.12 (0.37–3.37)
Obesity	22 (37.3)	2.11 (0.74–6.02)	**50 (84.7)**	1.98 (0.64–6.14)	21 (35.6)	0.86 (0.32–2.30)	**8 (13.6)**	5.21 (0.93–29.22)
BMI at W3								
Thin weight	31 (21.8)	0.86 (0.51–1.45)	96 (67.6)	0.99 (0.62–1.60)	44 (31)	0.89 (0.56–1.42)	6 (4.2)	0.83 (0.28–2.48)
Normal weight	254 (27.7)	Reference	642 (70)	Reference	375 (40.9)	Reference	55 (6)	Reference
Overweight	34 (28.3)	0.93 (0.51–1.72)	82 (68.3)	0.83 (0.46–1.48)	54 (45)	1.18 (0.68–2.04)	10 (8.3)	1.04 (0.35–3.11)
Obesity	21 (33.9)	0.75 (0.29–2.13)	50 (80.6)	1.12 (0.39–3.23)	26 (31.9)	1.02 (0.39–2.65)	6 (9.7)	0.49 (0.08–2.99)
ΔBMI-SDS W1-3		1.16 (0.83–1.61)		0.98 (0.71–1.35)		1.15 (0.85–1.55)		1.25 (0.68–2.28)

aOR represents adjusted odds ratio; CI, confidence interval; BMI-SDS, standardized score of body mass index; W1, Wave 1; W3, Wave 3. A significant difference in the χ2 linear-by-linear test for the association between variables of interest was marked in the bold type. Family monthly income was adjusted in multivariate binary logistic regression analysis.

**Table 4:** Association between the purposes of Internet use and pubertal timing and weight status among female adolescents.

	Chatting		Gaming		Non-academic browsing		Pornography viewing	
	N (%)	aOR (95% CI)	N (%)	aOR (95% CI)	N (%)	aOR (95% CI)	N (%)	aOR (95% CI)
Pubertal development								
Early puberty	78 (49.1)	1.22 (0.86–1.76)	27 (17)	0.59 (0.36–0.96)	94 (59.1)	1.07 (0.74–1.53)	2 (1.3)	NA
On-time puberty	378 (43)	Reference	190 (21.6)	Reference	490 (55.7)	Reference	2 (0.2)	NA
Late puberty	63 (41.7)	0.90 (0.61–1.32)	39 (25.8)	1.06 (0.68–1.64)	77 (51.0)	0.78 (0.54–1.14)	0 (0)	NA
BMI at W1								
Thin weight	67 (46.9)	1.34 (0.82–2.21)	33 (23.1)	0.54 (0.29–0.99)	77 (53.8)	0.82 (0.50–1.34)	0 (0)	NA
Normal weight	387 (43.4)	Reference	187 (21)	Reference	498 (55.8)	Reference	4 (0.4)	NA
Overweight	46 (44.2)	0.91 (0.50–1.65)	25 (24)	1.92 (0.96–3.83)	64 (61.5)	1.36 (0.75–2.49)	0 (0)	NA
Obesity	19 (38)	0.57 (0.19–1.65)	11 (22)	1.91 (0.52–1.94)	22 (44)	0.88 (0.31–2.54)	0 (0)	NA
BMI at W3								
Thin weight	51 (42.5)	0.71 (0.43–1.20)	36 (30)	2.50 (1.39–4.48)	64 (53.3)	0.98 (0.59–1.64)	1 (0.8)	NA
Normal weight	402 (43.7)	Reference	189 (20.6)	Reference	514 (55.9)	Reference	2 (0.2)	NA
Overweight	43 (43.9)	1.07 (0.58–1.98)	19 (19.4)	0.56 (0.26–1.20)	60 (61.2)	1.03 (0.55–1.92)	1 (1)	NA
Obesity	23 (44.2)	1.35 (0.45–4.07)	12 (23.1)	0.47 (0.12–1.81)	23 (44.2)	0.52 (0.17–1.55)	0 (0)	NA
ΔBMI-SDS W1-3		0.96 (0.66–1.30)		1.27 (0.87–1.86)		1.26 (0.91–1.74)		NA

aOR represents adjusted odds ratio; CI, confidence interval; BMI-SDS, standardized score of body mass index; W1, Wave 1; W3, Wave 3; NA, not applicable. Family monthly income was adjusted in multivariate binary logistic regression analysis. Due to an insufficient number of pornography viewers, regression analysis was not applicable to the female population

## Discussion

Our results demonstrate a link between pubertal timing and Internet use in an East Asian context, where research has been less explored in this aspect. Online pornography viewing was associated with early puberty in teenage boys, which came in line with previous studies using normative scores on the PDS to define pubertal status [14]. This was, however, inconsistent with those with measurements using perceived maturity in comparison with peers [21]. The explanation is that interpretations of perceived maturity likely vary according to personal perceptions that are formulated through the experiences of peer comparisons [22]. For example, viewing pornography could be regarded as a sign of maturity, hence a normative behavior among male peers [21]. Contrarily, this behavior was less practiced or less admitted among females, possibly due to cultural values or social undesirability [15]. Our study found that a very limited number of females reported pornography viewing. This may reflect a general prohibition against early romantic relationships and sexual debut in an East Asian context. On the other hand, early maturing girls were less likely to play online games; this was possibly due to the fact that they were more socially mature and thus less motivated by the playfulness of games [23]. From a practical perspective, the link between pubertal timing and exposure to pornography in male adolescents has its implications in the field of clinical care and public health. Previous research conducted in Taiwan has supported the claim that access to pornographic websites confers a potential risk of increased and early sexual behaviors [12, 24]. Moreover, frequent users of online pornography reported having unhealthier lifestyles, higher psychological distress, and more problematic behaviors [15, 25, 26]. What matters in viewing pornography at this age is that the adolescent brain might not be fully mature in terms of cognitive and emotional functions and judgments [27], thus endangering precocious individuals with a higher risk. Under the biological drives of gonadal hormones and neurobehavioral changes during puberty, early maturing adolescents may express higher interest in sexual behaviors [10]. Whether and how viewing pornography mediates the link between early sexual maturation and sexual activity requires more research.

Contrary to our expectation, obesity and increases in BMI did not contribute to Internet use. Thin-weight adolescents, instead, had a one-third increase in odds of Internet use. Our explanation for this discrepancy is that some other factors, for example physical activity and time for television watching and academic studies, were not measured in this cohort. Those with low Internet usage may remain inactive as they used spare time sitting studying or television watching. Moreover, in an academically competitive social context where nearly half of all students spend more than 2 hours in after-school tutorial classes [28], overtime Internet use is likely to restrict time for regular meals. Malnutrition or unbalanced nutrition in a growing adolescent may lead to weight problems, including underweight and obesity. A U-shaped relationship between Internet use and weight status during the third year of junior high school found in our results may reflect this argument, although the association at the obese end did not reach a significant level. Another explanation might be that pubertal timing is a strong and competitive predictor of Internet use in the regression analysis, so as to confound the link between weight status and Internet use.

Gender is another differentiating factor that warrants research attention. Thin weight was significantly associated with online game playing in females, whereas there was a higher proportion of obese males playing online games and viewing pornography websites, although the significance of these associations diminished after adjustment for sexual maturation. The implications of these findings could be two-fold. First, some websites, including online games, are designed to attract users by providing a communication and interaction forum. These Internet activities are highly rewarding, as adolescents spend more time and effort to get higher scores in the games and higher favors in the virtual network. Despite this, absorbance in the Internet may prevent them from engaging in other peer interactions and social activities in the real world, particularly for obese adolescent boys who may perceive themselves as less socially desirable because of their overweight status [29]. As such, a vicious cycle is likely formed if they continue to seek online sexually explicit materials designed to feed their sexual interests during the pubertal development [30]. Given that the Internet use at wave 3 was modeled as the outcome variable, weight status at earlier waves or changes of weight status may be considered causal factors in the inferential direction. Still, the causal relationship between weight status and Internet use for online gaming and pornography remains unanswered in this study, as we did not obtain the information regarding participants’ Internet use at baseline. Further research should be directed towards examining whether online gaming and pornography create obesogenic conditions or behaviors in adolescents.

This study has some limitations that need to be addressed. First, specific duration and intensity of Internet use for each purpose was not separately indicated in the survey. For example, we were unable to describe the exact hours of viewing pornography or playing games as only the overall Internet use was available. In this case, dose effect of BMI and pubertal status was not examined in the binary regression models. Moreover, some other contextual factors related to the Internet use, such as where (e.g. home or cyber café) and how (e.g. desktop computer or mobile electronic device) participants were connected to the Internet, were unavailable from the existing dataset of TYP. These factors are important in addressing adolescent behaviors of Internet use and should be further explored in future research. It is also worth of attention that technology and devices have greatly changed since the time of data collection. Adolescents currently are using more frequently emerging social media or websites, such as Facebook and Instagram, on their mobile phones or tablets. Analyzing old archived data have, however, been supported as this may generate new ideas or refine existing literature [31]. Readers should be cautions of this time gap, while further study with updated dataset is warranted to verify our findings. Lastly, social undesirability may have biased participants’ responses to sensitive questions, including those regarding online gaming and pornography. These online activities are generally perceived as nuisances to adolescents’ academic or behavioral development in Taiwan [32]. Despite this, online gaming is a common leisure use of the Internet among Taiwanese adolescents with approximately 5% reporting greater than 2 hours/day in another nationwide dataset [33]. As their age grows, high school students may become more permissive of online gaming and pornography viewing with nearly 70% reporting previous exposures to Internet pornography [34]. Having parents’ or teachers’ reports may ameliorate this sort of bias. However, parents may not accurately describe their children’s online activities. Obtaining encrypted big data regarding adolescent Internet use, upon their voluntary informed consent, from the end of Internet server may facilitate our understanding of users’ online behaviors.

In conclusion, early puberty was positively associated with Internet use in junior high school students. Particularly, early maturing adolescent boys had a higher likelihood of online pornography viewing. Given the potentially negative impacts of pornography exposure on subsequent sexual behaviors, appropriate health education should be provided to those who are at high risk. When weight status and pubertal timing were considered, sexual maturation came in as an important confounder in the link between the purposes of Internet use and weight status. Meanwhile, gender difference was noted in the association between adolescent weight status and Internet use. The causal directions of these associations should be revisited in future research before public health policy and guidance are tailored to be more culturally appropriate for this age group.

## Abbreviations

ANOVA: analysis of variance
BMI: body mass index
BMI-SDS: standardized BMI scores
CI: confidence interval
NTD: New Taiwan Dollar
OR: odds ratio
PDS: Pubertal Developmental Scale
PT: pubertal timing
TYP: Taiwan Youth Project
WS: weight status
